# The diagnostic value of RNA-mNGS and DNA-mNGS in differentiating bacterial infection from colonization in the lower respiratory tract

**DOI:** 10.3389/fcimb.2025.1639148

**Published:** 2025-09-09

**Authors:** Yuanfang Duan, Qin Li, Haitao Fei, Jiafu Song, Caiyun Xu

**Affiliations:** ^1^ Department of Respiratory and Critical Care Medicine, The First People’s Hospital of Lianyungang, Lianyungang, China; ^2^ Department of Critical Care Medicine, The First People’s Hospital of Lianyungang, Lianyungang, China

**Keywords:** RNA-mNGS, DNA-mNGS, sequencing reads, relative abundance, lower respiratory tract bacterial infection

## Abstract

**Background:**

Metagenomic next-generation sequencing(mNGS) has been widely used in the pathogenetic diagnosis of lower respiratory tract infections. However, the interpretation of pathogens detected by mNGS remains inconclusive.

**Objective:**

Our study aimed to compare the differential diagnostic value of sequencing reads and the relative abundance of bacteria detected by RNA-mNGS versus DNA-mNGS in distinguishing between bacterial infection and colonization in the lower respiratory tract.

**Methods:**

The hospitalized patients with suspected lower respiratory tract infections who had completed RNA-mNGS and DNA-mNGS testing at our hospital from June 2021 to December 2023 were reviewed and divided into two groups: the infected group and the colonized group, based on their final diagnoses. The Mann-Whitney U test was used to analyze differences in the number of bacterial sequencing reads and relative abundance between the two groups; the predictive capability of bacterial sequencing reads and relative abundance for identifying bacterial infections was evaluated using receiver operating characteristic (ROC) curves.

**Results:**

A total of 69 eligible patients were enrolled, with 85 detections of the four target bacterial species (*Pseudomonas aeruginosa*, *Acinetobacter baumannii*, *Klebsiella pneumoniae*, and *Corynebacterium striatum*) identified: 46 in infected patients and 39 in colonized patients. The number of sequencing reads and relative abundance of bacterial RNA and DNA in the pathogenic bacteria were significantly higher than those in the non-pathogenic bacteria (all *P*-values <0.01). ROC curves were used to evaluate the performance of the sequencing reads and relative abundance of bacterial species in predicting the responsible pathogens. The AUC value for RNA relative abundance was the highest at 0.991 (95% CI: 0.977-1.000, *P* < 0.001), with a cutoff value of 26.28%, a sensitivity of 0.957, and a specificity of 0.974. In the DNA-mNGS results, the AUC value for the ratio of the sequencing reads between the first and the second ranked bacterial sequences in predicting bacterial infection was [0.835 (95% CI: 0.742-0.928), *P* < 0.001], and the AUC value for the ratio of relative abundance in predicting bacterial infection was [0.839 (95% CI: 0.749-0.929), *P* < 0.001)], both having a cutoff value of 47.26, a sensitivity of 0.644 and a specificity of 0.929.

**Conclusions:**

Bacterial relative abundance and sequencing reads can serve as indicators to distinguish between infection and colonization, and the relative abundance based on RNA-mNGS exhibits the best differential diagnostic performance; when DNA-mNGS results stand alone, the relative abundance of the detected bacteria and the ratio of relative abundance between the first-ranked and the second-ranked detected bacteria can be utilized for a comprehensive assessment of infection versus colonization.

## 1 Introduction

Lower respiratory tract infections (LRTIs) are prevalent worldwide, especially in children, the elderly, and immunocompromised individuals. There are numerous pathogens that cause LRTIs, including bacteria, fungi, and viruses (Torres et al., 2021). However, the respiratory tract is not a sterile lumen but rather possesses a rich microbiome with numerous colonizing bacteria, In immunocompromised patients, nearly all bacterial species may potentially serve as pathogens for lung infections (De La Cruz and Silveira, 2017).Inadequate or excessive antimicrobial therapy may lead to adverse outcomes or even endanger patients survival. Therefore, it is particularly important to distinguish between true pathogens causing infection and colonizers. Traditional bacterial testing methods, including smear microscopy, microbial culture, PCR, and serum antibody testing, have such low positive rates that a pathogenetic diagnosis is not obtained in approximately 50% of patients with community-acquired pneumonia (Torres et al., 2021; Zheng et al., 2021). In traditional detection methods, for sterile specimens (e.g., blood, tissue, bone marrow, serous cavity fluid), a positive bacterial culture may confirm the etiological diagnosis. However, for open respiratory tract specimens, a positive bacterial culture still requires differentiation between infection and colonization. First, specimen quality must be assessed: bronchoalveolar lavage fluid (BALF) is of higher quality than sputum specimens. Second, quantitative culture methods can be applied for differentiation: when the bacterial concentration in BALF culture is ≥10^4^ CFU/mL or that in protected brush specimens is ≥10³ CFU/mL, the likelihood of pathogenic bacteria being present increases. Finally, close integration with clinical context is essential, including evaluation of high-risk factors, host immune status, relevant clinical symptoms and signs, and efficacy after adjustment of the treatment regimen (Fagon et al., 2000; Torres and Ewig, 2004; Rea-Neto et al., 2008). In hospital-acquired pneumonia and ventilator-associated pneumonia (Torres et al., 2021), *Pseudomonas aeruginosa*, *Klebsiella pneumoniae*, and *Acinetobacter baumannii* are frequently detected, but their colonization is also common in clinical practice. *Corynebacterium striatum* is widely found on human skin and in the respiratory tract and is a conditionally pathogenic bacterium. It is currently considered one of the causative agents of severe LRTIs (Silva-Santana et al., 2021). Physicians often struggle to reliably differentiate between infection and colonization for the four target bacterial species detected in lower respiratory tract specimens, while culture-based diagnostics—time-consuming by nature—fail to provide timely guidance for initial therapy.

Metagenomic next-generation sequencing(mNGS) technology is currently widely used in the pathogenetic diagnosis of LRTIs due to its rapidity, efficiency and sensitivity, mNGS is an unbiased approach to detect the DNA and RNA of pathogens in a clinical sample (Gu et al., 2019). However, there is still no consensus on how to differentiate between infection and colonization for pathogens detected by mNGS. Therefore, it is necessary to establish a rapid and accurate method for identifying pathogens detected by mNGS. Pathogen sequencing reads and relative abundance are two important indicators in mNGS reporting. Sequencing reads is the number of pathogen genomes detected by mNGS, which positively correlates with the quantity of the microorganism in the specimen (Chinese Society of Bacterial Infection and Drug Resistance Prevention, 2022).; Relative abundance refers to the proportion of the microorganism’s genome within its corresponding classification (four categories: bacteria, fungi, viruses, and parasites) after excluding host sequences; the higher the abundance, the higher the proportion of the microorganism (Expert Consensus Group on the Application of Macrogenomic Analysis and Diagnostic Techniques to Acute and Critical Infections, 2019). Liu et al. found that the sequencing reads and relative abundance of bacterial sequences detected by DNA-mNGS can be used to predict whether the detected bacteria represent true pathogens causing infection or colonizers (Liu et al., 2022). Wang et al. found that the DNA sequencing reads in fungal infections can better predict infection and colonization (Wang et al., 2022). However, because RNA represents the transcriptional level of DNA, the detection of DNA can only indicate what kind of organism exists, and the detection of RNA can reveal that this organism has transcriptional activity (De La Cruz and Silveira, 2017; Emerson et al., 2017). The responsible microorganism may be more active and produce more transcripts than the colonized microorganism (Zhao et al., 2021), and the transcripts of DNA organisms can also be detected through the RNA workflow. Based on the aforementioned theory, our previous research has shown that compared to DNA-mNGS, RNA-mNGS reduced the misdiagnosis rate of bacterial pathogens in LRTIs (Song et al., 2024). This study aimed to explore whether the reads and relative abundance of bacterial sequences, detected by DNA-mNGS and RNA-mNGS, could be used to distinguish between infection and colonization in patients with common clinical bacterial detections, and to compare whether there were any differences between the two methods.

## 2 Materials and methods

### 2.1 Participants

This retrospective study included a total of 69 patients with suspected LRTIs, who were hospitalized at the First People’s Hospital of Lianyungang from June 2021 to December 2023. Patients were enrolled according to the guidelines for the management of LRTIs in adults of the European Respiratory Society and the European Society for Clinical Microbiology and Infectious Diseases (Woodhead et al., 2011). Enrolled patients were required to complete both DNA-mNGS and RNA-mNGS, having at least one of the four species detected: *P. aeruginosa, A. baumannii, K. pneumoniae*, and *C. striatum*. In this study, a total of 69 eligible hospitalized patients were enrolled and the specimens sent for testing were BALF.

Informed consent was obtained from all patients or their legal guardians. The study was approved by the Ethics Committee of The First People’s Hospital of Lianyungang (Identifier: LW-20241202001-01) and was carried out in accordance with the tenets of the Declaration of Helsinki.

### 2.2 Specimen collection and processing

Bronchoscopic alveolar lavage was completed in 69 patients, and 10–20 ml of BALF specimens were collected according to standard procedures (Levy et al., 2018). After sampling, the specimens were divided into two portions, one was sent to the laboratory of our hospital for conventional microbiological detection, and the other was immediately put into mNGS sequencing EP tubes containing DNA/RNA Shield™ (Zymo Research, USA), and then transported to the laboratory (Dinfectome Inc., Nanjing, China) for subsequent nucleic acid extraction and sequencing analysis. The sampling and retention of samples were both conducted by trained, dedicated personnel.

### 2.3 Nucleic acid extraction

For patients’ BALF specimens, DNA was extracted using the TIANamp Magnetic DNA Kit (TIANGEN, China) according to the manufacturer’s protocol. The quality and quantity of extracted DNA were measured using Nanodrop 8000 spectrophotometers and Qubit 2.0 Fluorometers Nanodrop (Thermo Fisher Scientific, USA). RNA was extracted from the supernatant using the QIAamp Viral RNA Mini Kit (QIAGEN, Hilden, Germany).

### 2.4 Library preparation and sequencing

DNA sequencing libraries were prepared using the Hieff NGS C130P2 OnePot II DNA Library Prep Kit for MGI (Yeasen Biotech, Shanghai, China) according to the manufacturer’s instructions. For RNA library preparation, ribosomal RNA (rRNA) was removed from total RNA using the Hieff NGS MaxUp rRNA Depletion Kit (Yeasen Biotech, Shanghai, China). In this process, rRNA-specific probes within the kit selectively hybridized with rRNA, forming DNA-RNA heteroduplexes that were subsequently degraded and removed by RNase H. The resulting rRNA-depleted RNA was then subjected to reverse transcription and strand-specific library construction using the Hieff NGS RNA Library Prep Kit (Yeasen Biotech, Shanghai, China) to generate the final RNA library. The quality control was performed using 2100 Agilent High Sensitive DNA chips (Agilent, Santa Clara, CA, USA), and libraries were sequenced in the single-end 50 bp sequencing mode using MGISEQ-200 (MGI Technology, Shenzhen, China).

### 2.5 Sequencing data processing

We use an in-house developed bioinformatics pipeline for pathogen identification. Briefly, adapter contamination, duplicate reads, low-quality reads, and short reads (length<36bp) were removed from the raw sequencing data to generate high-quality data. Human host sequences were identified by mapping to the human reference genome (hs37d5) using Bowtie2 (version 2.2.6). To identify pathogens, reads that were unable to be mapped to human genomes were retained and aligned against a microbial genome database. A customized local microbial genomic database was constructed by integrating all available genome assembly data of infectious pathogens from the NCBI GenBank (data accessed in April 2021). This database comprises genomic sequences from 24,614 pathogenic species, including bacteria, fungi, viruses, and parasites. A total of 48,911 genome assemblies were obtained, including 26,271 at the “complete genome” level, 8,881 at the “scaffold” level, 11,408 at the “contig” level, and 2,351 at the “chromosome” level. These data were subsequently used for comparative genomic analyses.

### 2.6 Interpretation and reporting

The mNGS pathogen detection pipeline was described in previous studies (Zeng et al., 2022), and the criteria for a positive detection are as follows: 1. at least one species-specific read for *Mycobacterium*, *Nocardia*, and *Legionella pneumophila* detection; 2. for bacteria (excluding *Mycobacterium*, *Nocardia* and *Legionella pneumophila*), virus, parasites and fungi, the result was considered positive if a species detected by mNGS had at least three non-overlapping reads; 3. If the ratio of microorganism reads per million in a given sample to those in the negative ‘no-template’ control(NTC) is<10,the pathogens are excluded.

### 2.7 Diagnosis of lower respiratory tract infections

The final diagnosis of LRTIs in 69 patients was established after comprehensive evaluation by two chief respiratory physicians, discrepant results were adjudicated by a third expert. Assessment involves: 1. Patient’s baseline immune status and bacterial infection-related risk factors (presence/absence).​2. The patient’s clinical symptoms; 3. Chest CT or X-ray findings;4. Traditional microbiologic tests, mNGS, complete blood count (CBC), C-reactive protein(CRP), procalcitonin (PCT), and other indicators of infection, as well as serological examinations [including fungal (1-3)-β-D glucan test, serum cryptococcal capsular polysaccharide antigen test, and Mycoplasma pneumoniae serological antibody detection].5.Re-evaluation and correction of the final diagnosis based on the patient’s clinical outcomes following treatment. Bacteria detected by conventional testing or mNGS were considered true positives (infected group) only if they were consistent with the final clinical diagnosis; otherwise, they were considered false positives (colonized group).

### 2.8 Statistical methods

Data were analyzed using SPSS 26.0 statistical software. Data were expressed as mean ± standard deviation (SD) or median (interquartile spacing) M (IQR); Mann-Whitney U test was used for intergroup comparisons, and Pearson correlation analysis was used for correlation analyses. The diagnostic performance of bacterial DNA and RNA sequencing reads and relative abundance was evaluated using receiver operating characteristic curve (ROC curve), and the optimal critical value was calculated. A *P* value of <0.05 was considered statistically significant.

## 3 Results

### 3.1 Participants’ baselines

**Figure 1:**
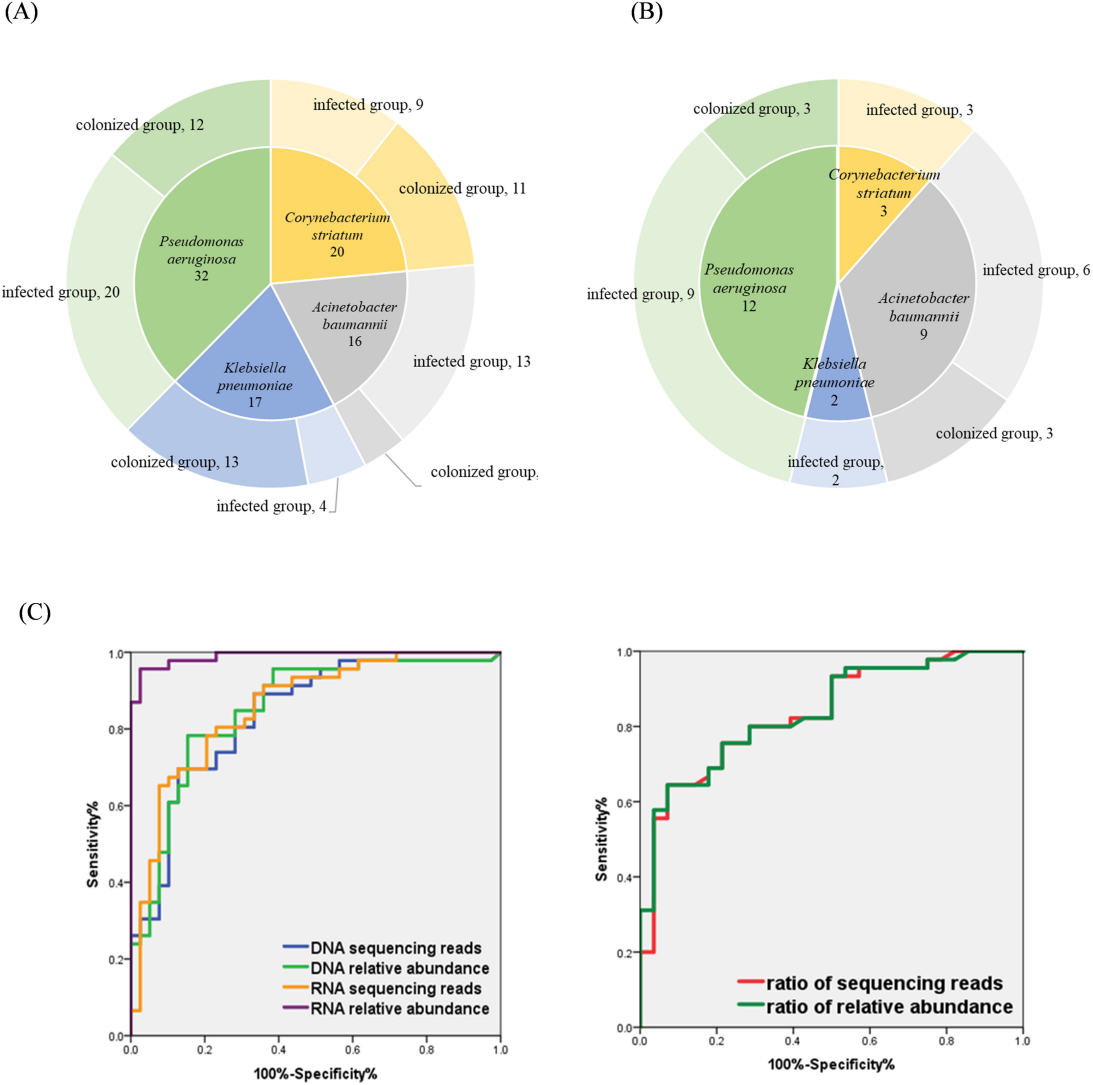
**(A)** Distribution of the four bacterial species in mNGS results; **(B)** Distribution of four bacterial species in conventional culture results; **(C)** Evaluating the performance of indicators to identify bacterial infection and colonization using ROC curves.

A total of 69 hospitalized patients with detection of *P. aeruginosa*, *A. baumannii*, *K. pneumoniae*, and *C. striatum* by mNGS were collected, of which 46 (67%) were male and 23 (33%) were female. The patients were mainly from the respiratory department (48 cases, 70%), while the rest were from the geriatrics department (6 cases, 8%) and the intensive care unit (ICU) (15 cases, 22%). There were 13 patients (18.84%) who were immunocompromised, 11 patients (15.94%) with underlying lung disease, 9 patients (13.04%) with risk of aspiration, 2 patients (2.90%) with open airway, and 17 patients (24.64%) evaluated with severe pneumonia, and the specific baseline characteristics are shown in **Table 1**. Of the 69 BALF specimens were completed with both RNA-mNGS and DNA-mNGS. A total of 85 times of the above four bacteria were detected by mNGS, including 32 samples with *P. aeruginosa*, 16 samples with *A. baumannii*, 17 samples with *K. pneumoniae*, and 20 samples with *C. striatum.* Following comprehensive expert evaluation, 46 detections of the four target bacteria were identified in samples from infected patients, whereas 39 detections were recorded in samples from the colonized patients (see **Figure 1A**), with a true positive rate of 66.67%. 69 specimens were sent for traditional bacterial culture at the same time, and a total of 26 times of the four target bacteria were detected, with a detection rate of 37.68%, of which 20 times from infected patients and 6 times from colonized patients (see **Figure 1B**), with a true positive rate of 28.99%.

**Table 1:** Baseline characteristics of the sample.

Characteristics	Value
Patients, total(n)	69
Age (year)	70.07 ± 16.44
Gender, n (%)	Male, 46 (67%), Female, 23(33%)
Department	RES 48, ICU 15, GER 6
Immunocompromised	13 (Long-term use of steroids in 6 cases, type 2 diabetes in 4 cases, 3 cases after chemotherapy)
underlying lung disease	11 (3 cases of COPD, 8 cases of bronchiectasis)
risk of aspiration	9
open airway	2
severe pneumonia	17
Strains detected by mNGS	85 strains (46 from infected group, 39 from colonized group)
Strains detected by traditional bacterial culture	26 strains (20 from infected group, 6 from colonized group)

RES, respiratory department; ICU, intensive care unit; GER, geriatrics department; COPD, chronic obstructive pulmonary disease.

### 3.2 Comparison of the differences in the four indicators (DNA sequencing reads, DNA relative abundance, RNA sequencing reads, RNA relative abundance) between the infected group and the colonized group

Statistical results showed:1. In the infected group, the number of bacterial RNA sequencing reads, RNA relative abundance, the number of DNA sequencing reads, and DNA relative abundance were all significantly higher than those in the colonized group, with statistically significant differences (all *P*-values < 0.001, see **Table 2**). 2. Within the infected group, for the same bacterial species, both the number of RNA sequencing reads and relative abundance were higher than those of DNA detection, though no statistically significant differences were observed (all *P*-values > 0.05). In contrast, within the colonized group, RNA relative abundance was significantly lower than DNA relative abundance, and the number of RNA sequencing reads were significantly lower than the number of DNA sequencing reads, with statistically significant differences (all *P*-values < 0.05; **Tables 3**, **4**).

**Table 2:** Comparison of the four indicators between the infected and colonized groups [M (IQR)].

Variable	RNA relative abundance	RNA sequencing reads	DNA relative abundance	DNA sequencing reads
Infected group	87.09% (30.58%)	58306 (177039)	93.69% (36.45%)	51591 (197451)
Colonized group	2.97% (12.82%)	424 (3710)	13.37% (48.82%)	1417 (12673)
Z-statistic	-7.761	-5.653	-5.578	-5.322
*P*- value	0.000	0.000	0.000	0.000

**Table 3:** Comparison of RNA relative abundance with DNA relative abundance [M (IQR).

Variable	Infected group	Colonized group
RNA relative abundance	87.09% (30.58%)	2.97% (12.82%)
DNA relative abundance	93.69% (36.45%)	13.37% (48.82%)
Z-statistic	-7.727	-3.053
*P*- value	0.468	0.002

**Table 4:** Comparison of RNA sequencing reads with DNA sequencing reads [M (IQR)].

Variable	Infected group	Colonized group
RNA sequencing reads	58306 (177039)	424 (3710)
DNA sequencing reads	51591 (197451)	1417 (12673)
Z-statistic	-1.196	-1.982
*P*-value	0.232	0.048

### 3.3 ROC curve analysis for distinguishing infection vs. colonization

With the final clinical diagnosis as the gold standard, ROC curves were used to assess the performance of sequencing reads and relative abundance of each bacterial species in distinguishing between infection or colonization. As shown in **Figure 1C** and **Table 5**: The area under the curve (AUC) of bacterial RNA relative abundance was 0.991 (95%CI:0.977-1.000), *P*<0.001; the cutoff value was 26.28%, the sensitivity was 0.957, and specificity was 0.974. The AUC of bacterial RNA sequencing reads was 0.857 (95%CI:0.777-0.938), *P*<0.001; the cutoff value was 4506, the sensitivity was 0.783, and specificity was 0.795. The AUC of bacterial DNA relative abundance was 0.853 (95%CI:0.969-0.936), *P*<0.001; the cutoff value was 56.07%, the sensitivity was 0.783, and specificity was 0.846.The AUC of bacterial DNA sequencing reads was 0.836 (95%CI:0.750-0.923), *P*<0.001; the cutoff value was 21029, the sensitivity was 0.696, and specificity was 0.872.

**Table 5:** Comparison of AUC values.

Variable	AUC values (95%CI)	Cutoff value	Sensitivity	Specificity	Youden
RNA relative abundance(n=85)	0.991(0.977-1.000)	26.28%	0.957	0.974	0.931
RNA sequencing reads(n=85)	0.857(0.777-0.938)	4506	0.783	0.795	0.578
DNA relative abundance(n=85)	0.853(0.969-0.936)	56.07%	0.783	0.846	0.629
DNA sequencing reads(n=85)	0.836(0.750-0.923)	21029	0.696	0.872	0.568
Ratio of DNA sequencing reads (n=73)	0.835(0.742-0.928)	47.26	0.644	0.929	0.573
Ratio of DNA relative abundance (n=73)	0.839(0.749-0.929)	47.26	0.644	0.929	0.573

### 3.4 Sequencing reads and abundance ratios in DNA-mNGS ROC curves: distinguishing infection vs. colonization

In the results of DNA-mNGS testing, the species with the highest number of sequencing reads and relative abundances were further selected, which were one of the following four species: *P. aeruginosa*, *A. baumannii*, *K. pneumoniae*, and *C. striatum.* In this section, a total of 81 patients were enrolled, comprising 69 previously described patients and 12 additional patients who underwent DNA- mNGS alone. Of the 81 samples, 73 (with the highest sequencing read counts and relative abundances attributed to one of the four target species) were further categorized: 45 were identified infection and 28 were identified colonization based on the final clinical diagnosis determined by experts. Statistical analysis showed that in the infected group, the ratio of reads of the first- ranked bacteria to the second-ranked bacteria was significantly higher than in the colonized group, and the relative abundance ratio was also significantly higher (all *P*-values<0.0001, see **Table 6**). The performance of sequencing reads ratio and relative abundance ratio in differentiating between bacterial infection or colonization was evaluated using ROC curves. The results showed that the AUC of the sequencing reads ratio was 0.835 (95%CI:0.742-0.928), *P* <0.001. The cutoff value was 47.26, the sensitivity was 0.644, the specificity was 0.929. The AUC of the relative abundance ratio was 0.839 (95%0.749-0.929), *P*<0.001. The cutoff value was also 47.26, the sensitivity was 0.644, the specificity was 0.929 (see **Table 5**).

**Table 6:** Comparison between two groups of sequencing reads ratios and relative abundance ratios in the DNA-mNGS [M (IQR)].

Variable	ratio of sequencing reads *	ratio of relative abundance *
Infected group	181.80 (2165.77)	296.79 (9713.16)
Colonized group	2.77 (13.26)	2.78 (13.27)
Z-statistic	-4.788	-4.844
*P*-value	0.000	0.000

* Ratio of sequencing reads: the ratio of sequencing reads between the strains ranked first and second in terms of sequencing reads; Ratio of relative abundance: the ratio of relative abundance between the strains ranked first and second in terms of relative abundance.

## 4 Discussion

The results of this study indicate that the number of bacterial sequencing reads and relative abundance can better distinguish between bacterial infection and colonization, among which the relative abundance of RNA is the best indicator. When the detected relative abundance of bacterial RNA is > 26.28%, the sensitivity for identifying bacterial infection (vs. colonization) is 0.957, and the specificity is 0.974.

Given that RNA represents the transcription level of DNA, and the responsible pathogen is more active than the colonized one, producing more transcripts, theoretically, the RNA-metagenomic next-generation sequencing (RNA-mNGS) approach is more likely to detect the responsible pathogen. Chen L et al.’s research has confirmed that using RNA-mNGS alone can accurately identify the responsible pathogen (Chen et al., 2020). Zhao N et al. used meta-transcriptomics using third-generation sequencing (mtTGS) found that RNA can be used as a target molecule for microbial analysis and can be applied to the identification of pathogens in clinical samples, and its sequencing efficiency exceeds that of DNA-based mNGS detection (Zhao et al., 2021). The above mentioned research is consistent with our findings. Our research has found that the DNA sequencing reads and their relative abundance, as well as RNA sequencing reads, and their relative abundance in the infected group are significantly higher than those in the colonized group. Moreover, within the infected group, there is no statistical difference between RNA and DNA; while within the colonized group, the reads and relative abundance of RNA are significantly lower than those of DNA. This indicates that the reads and relative abundance of RNA are significantly superior to DNA in distinguishing between bacterial infection and colonization. However, Zhao N’s research focuses on identifying pathogens in clinical samples rather than identifying responsible pathogens. More importantly, the above two studies failed to clarify the cutoff values of the reads and relative abundance, and their clinical guiding significance requires further investigation and improvement. And our research precisely addresses this deficiency. However, due to the susceptibility of RNA to degradation, the RNA-mNGS detection process places relatively stringent requirements on specimen collection, preservation, and transportation conditions. To solve this problem, all sample detections in this study were carried out in Difei Laboratory (Dinfectome Inc., Nanjing, China), and a nucleic acid protectant: DNA/RNA™ Shield (Zymo Research, USA) was used during sample collection and transportation, which effectively avoided the degradation of RNA and had no adverse effects on the subsequent detection process (Bell et al., 2023).

Although the sequencing reads and relative abundance of DNA-mNGS are less effective than RNA-mNGS in differentiating between bacterial infection and colonization, combined detection of RNA and DNA is costly and challenging to implement widely in clinical practice. Our study found that using only the ratio of sequencing reads and the ratio of relative abundances of the first - and second - ranked detected bacteria in the DNA-mNGS detection results can also effectively distinguish bacterial infection from colonization. When the ratio of the two was >47.26, the specificity for predicting the first ranked detected organism as the responsible pathogen was 0.929 while the sensitivity was 0.644, slightly lower. This may be related to the rapid formation of the “occupancy effect” following the invasion of the responsible pathogen. Responsible pathogens are favored in space and nutrient resources through exploitative and disruptive competition, thus inhibiting the growth of other bacteria groups (Ghoul and Mitri, 2016). In addition, our study found that DNA relative abundance has superior diagnostic value in differentiating bacterial infection from colonization compared to sequencing reads. Chen Tet al. found that when using DNA-mNGS to detect bronchial aspirate specimens, the differential diagnostic value of relative abundance is better than that of the sequencing reads (Chen et al., 2023), which is consistent with our study. In order to remove the impact of DNA-mNGS sequencing depth and gene length on different pathogens, Liu H et al. used Ig(RPKM) (Reads Per Kilo-base per Million reads) instead of the normalized sequencing reads, and they found that normalized sequencing reads were superior to relative abundance in the identification of the responsible pathogens (Liu et al., 2022), which is in contrast to the present study. We consider that the sequencing reads is affected by a variety of factors, such as the sequencing depth and gene length, as well as the amount of sequencing data, the proportion of human - derived data, the size of the species genome and the proportion of genome-specific sequences. Therefore, there will be large differences in the sequencing reads reported by different laboratories, whereas relative abundance may offer better comparability. In summary, we propose that for the interpretation of results using only the DNA-mNGS process, relative abundance and relative abundance ratios can be comprehensively assessed to identify the responsible pathogens to improve accuracy.

The four bacteria included in this study are common in LRTIs both in terms of infection and colonization. Among them, *P. aeruginosa, K. pneumoniae*, and *A. baumannii* are the main causative agents in hospital-acquired pneumonia and ventilator-associated pneumonia worldwide (Pulmonary Infection Assembly of Chinese Thoracic Society, 2018; Torres et al., 2021). In community-acquired pneumonia, a global study showed that *P. aeruginosa* and *K. pneumoniae* were the most common pathogens other than *S. pneumoniae*, with detection rates of 4.1% and 3.4% (Carugati et al., 2020). The detection rate of *P. aeruginosa* can reach up to 8.3% in patients with severe community-acquired pneumonia and up to 67% in patients with bronchiectasis, very severe chronic obstructive pulmonary disease, and tracheotomy (Cilloniz et al., 2016b; Restrepo et al., 2018). *C. striatum* is a Gram-positive bacillus that is widely hosted in the human skin and respiratory tract as a conditionally pathogenic organism. A worldwide investigation has revealed that it is a potentially multidrug-resistant (MDR) pathogenic microorganism susceptible to causing serious infections in patients with prolonged hospitalization, history of repeated antibiotic use, undergone invasive procedures, or immune compromise (Silva-Santana et al., 2021). However, previous literature reported that the positivity rate of traditional detection methods for lower respiratory tract bacterial infections was 8.3%-47.2% (Zheng et al., 2021), and the positive rate of traditional bacterial culture in our study was 37.68%, which is consistent with previous studies. mNGS significantly increased the bacterial detection rate, but on the other hand, the false-positive rate of mNGS was also high, especially for these bacteria that are easily colonized. However, most of the current studies still focus on the comparison of the positive rate of mNGS with traditional detection methods (Zheng et al., 2021; Sun et al., 2022), with less emphasis on how to identify bacterial infection and colonization from mNGS results. Moreover, no study has yet explored the role of RNA expression from microorganisms detected by DNA-mNGS in distinguishing between bacterial infection and colonization. Our study addresses this gap and provides a feasible approach using integrated DNA and RNA mNGS analysis for interpreting results.

The present study has limitations. First of all, we only analyzed the above four bacteria, and the differential diagnostic performance of RNA-mNGS sequencing reads and relative abundance in other microorganisms need further investigation through ongoing sample accumulation. Especially for pathogens that are difficult to extract nucleic acids: Mycobacterium (including TB and NTM), fungi (*Aspergillus, Cryptococcus*, etc.), and intracellularly growing pathogens such as *Chlamydia, Rickettsia, Orientia*, and *Coxiella*. Secondly, in the DNA-mNGS process, the sequencing reads ratio and relative abundance ratio are used to identify the responsible pathogens, which theoretically ignores the situation of mixed bacterial infections, leading to high specificity but compromised sensitivity. Epidemiologic data from Europe and the United States show that mixed infections account for 5-6% of community-acquired pneumonia (Torres et al., 2021), with the common types of mixing being: two bacterial infections in 32%, mixed bacterial-viral infections in 29%, and mixed bacterial and atypical pathogen infections in 18% (Cilloniz et al., 2016a). A study that included 256 cases of hospital-acquired pneumonia found that the percentage of mixed infections was 16%, mostly mixed bacterial infections (Ferrer et al., 2015). Therefore, overall mixed bacterial infections in pneumonia remain a rare pattern of infection.

In conclusion, our study revealed that the RNA-mNGS was superior to DNA-mNGS in identifying bacterial infection versus colonization caused by common bacteria in the lower respiratory tract (*P. aeruginosa, K. pneumoniae, A. baumannii, and C. striatum*), with RNA relative abundance being the optimal indicator. When using DNA-mNGS alone, it is recommended to use a combination of bacterial relative abundance and relative abundance ratio between the top two ranked bacteria to differentiating between bacterial infection and colonization.

## Data Availability

The datasets presented in this study can be found in online repositories. The raw sequence data reported in this paper have been deposited in the Genome Sequence Archive in National Genomics Data Center, China National Center for Bioinformation / Beijing Institute of Genomics, Chinese Academy of Sciences (GSA: CRA028763) that are publicly accessible at https://ngdc.cncb.ac.cn/gsa.
